# Abdominal Cocoon Syndrome (Idiopathic Sclerosing Encapsulating Peritonitis): How Easy Is Its Diagnosis Preoperatively? A Case Report

**DOI:** 10.1155/2013/604061

**Published:** 2013-05-07

**Authors:** Julius A. A. Awe

**Affiliations:** Department of Surgery, College of Health Sciences, Igbinedion University, Okada, Private Bag 0006, Edo State, Nigeria

## Abstract

The abdominal cocoon syndrome (or idiopathic encapsulating peritonitis) is a rare cause of intestinal obstruction. It has been reported predominantly in adolescent girls living in tropical/subtropical region in which diagnosis is only made at laparotomy in most cases. The cause and pathogenesis of the condition have not been elucidated. Prolonged administration of practalol, meconium peritonitis, and tuberculous infection of the female genital tract have been incriminated as possible causes. The author reports a case of a female patient with recurrent intestinal obstruction treated for years but failed to settle down on conservative treatment during her last hospital admission and had to undergo surgery. Preoperative diagnosis of this syndrome as the cause of her intestinal obstruction was not made until at laparotomy, when a thick fibrotic peritoneal wrapping of the bowel in a concertina-like fashion with some adhesions was found. Excision of this membrane and adhesiolysis were carried out without any need for bowel resection, and this led to relief of the obstruction and patient's complete recovery. Awareness of this benign condition in the differential diagnosis of intestinal obstruction will result in early diagnosis and correct management and prevent unnecessary bowel resections and bad outcomes.

## 1. Introduction

Abdominal cocoon syndrome (or idiopathic encapsulating peritonitis) is a relatively rare cause of intestinal obstruction, described mostly in young adolescent girls, and was first described in 1978 [1].

The etiology of this disease is largely unknown and the cases seen so far have been limited to the tropical and subtropical zones and primarily affect young adolescent females, even though several earlier cases have been reported also in males [2, 3].

Thick fibrotic peritoneum encasing the small bowel partially or completely is a pathognomonic feature, and the correct diagnosis is not often made preoperatively.

This is the case report of an eighteen- (18-) year-old Nigerian girl that presented with intestinal obstruction [4] that failed to settle down on conservative treatment during her last hospital admission and had to undergo surgery.

The author is therefore presenting the case so that practicing surgeons have better awareness of this condition as a possible cause in the differential diagnosis of intestinal obstruction which may facilitate pre-operative diagnosis, prevent inadvertent bowel damage at laparoscopy, and prevent unnecessary bowel resection at laparotomy [5].

## 2. Case Report

An eighteen- (18-) year-old Nigerian girl presented with severe central/lower abdominal pain associated with a few episodes of bilious vomiting which started about two (2) to three (3) days at home before admission to the hospital.

She took some self-medication at home which is a common practice in this part of the developing world, but unfortunately her condition did not improve.

Clinical examination revealed a moderately ill-looking slightly dehydrated young lady. Temperature 37.8°C; BP 100/70 mmHg; Pulse 80/min; RR 24/min. Abdomen was tender and moderately distended at its central/lower part.

A palpable mass at the right lower paraumbilical area was found. Bowel sounds were found to be sluggish.

Significant in her past medical is the fact that she had recurrent antibiotic therapy for the past three years following an undisclosed intravaginal medical treatment by a local quack practitioner.

**Figure 1:**
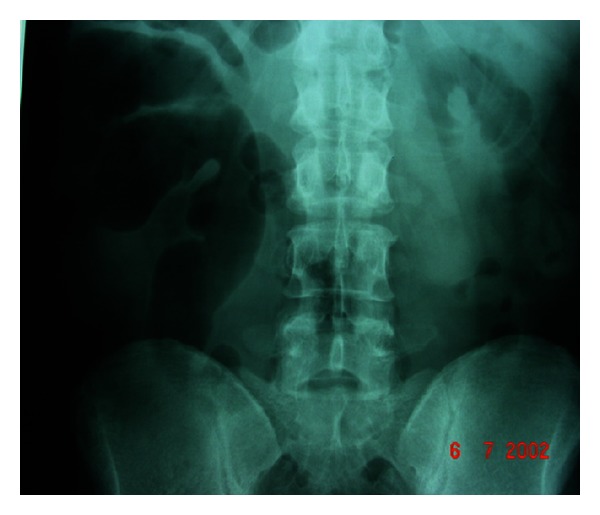


**Figure 2:**
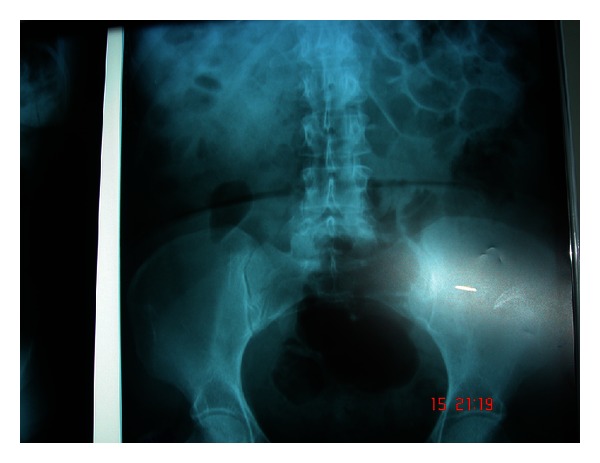


Plain abdominal X-rays were unremarkable (see Figures 1 and 2).

Total white cell count and Hemoglobin were within normal limits.

A provisional diagnosis of acute-on-chronic intestinal obstruction was made.

She was therefore placed on the usual regimen of nil by mouth, intravenous fluid therapy, and nasogastric tube suction.

Patient continued to be monitored on the ward, but her abdominal pain and distention got worse with increased bilious output from the nasogastric tube.

A decision was then taken to carry out an exploratory laparotomy on her.

**Figure 3:**
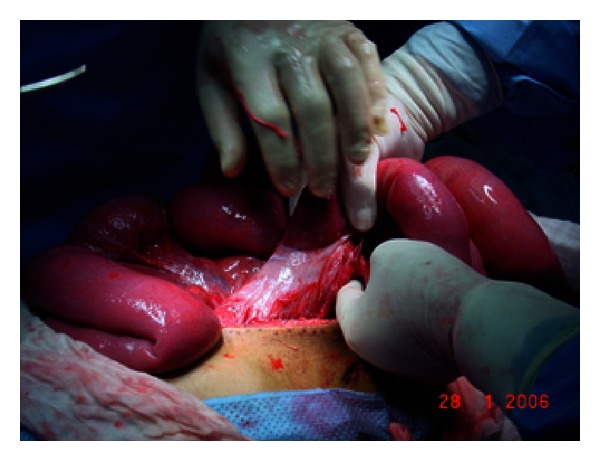
Displaying part of the adhesions.

**Figure 4:**
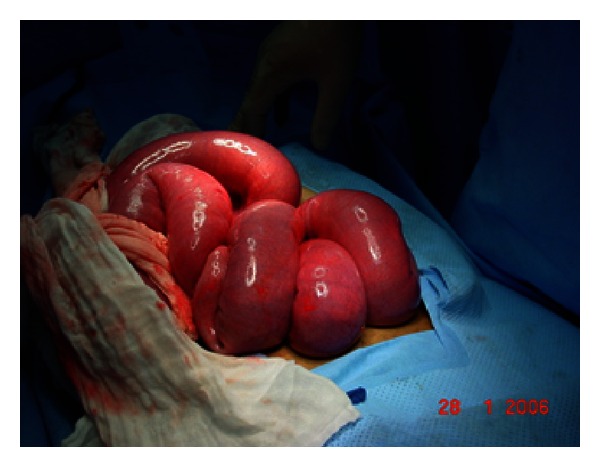
Showing some freed bowel.

At laparotomy a thick fibrotic peritoneum wrapping the bowel in a concertinalike fashion with some adhesions extending almost to the pelvic brim was found. Total careful excision of this membrane and lysis of adhesions were carried out and the underlying bowel was found to be quite healthy that needed no resection (see Figures 3 and 4).

Postoperative period was uneventful and the patient was discharged home after one week and discharged from surgical outpatient followup after one (1) month.

## 3. Discussion and Conclusion

Abdominal cocoon syndrome (or idiopathic encapsulating peritonitis) is a rare disease of the peritoneum and almost invariably presents as an acute or subacute intestinal obstruction with or without a mass, which is usually diagnosed incidentally at laparotomy [6].

It was first described by Foo et al. in 1978 [1]. Characteristically the bowel is found totally or partially coiled up in a concertina fashion under the encased thick fibrous white membrane and still up to date of an unknown aetiology [7, 8].

Some authors have implicated prolonged administration of practalol therapy as a possible aetiology in some cases, meconium peritonitis, sarcoidosis, orthotopic liver transplantation, indwelling abdominal catheters, or even tuberculous infection of the female genital tract [9].

These conditions may predispose patients to chronic peritoneal irritation and inflammation, which as a final effect leads to peritoneal fibroneogenesis.

Cell-mediated immunological tissue damage initiated by microorganisms assessed by immune-fluorescent studies has been documented in some cases as possible cause. Pre-operative tissue culture of peritoneal membrane may also contribute in future in the further evaluation of the aetiology of abdominal cocoon syndrome [6]. 

In this patient, it was probably secondary to her previous intravaginal treatment a couple of years prior to her presenting with recurrent lower abdominal pain and subacute intestinal obstruction. 

To the best of the author's knowledge, this the second recorded case in Nigeria [10]. She could have developed secondary peritonitis arising from her previous genital treatment that resulted in the formation of the adhesions that eventually formed to plaster the bowel.

Despite anecdotal reports of a preoperative diagnosis especially by medical imaging, in the majority of cases of idiopathic encapsulating peritonitis, it is a fortuitous finding. A better awareness of this condition is what may facilitate pre-operative diagnosis [11, 12].

A high index of clinical suspicion [13, 14], with good history detailing some of the previously mentioned associated causes combined with relevant imaging findings [15, 16], is recommended in enhancing pre-operative diagnosis of abdominal cocoon syndrome.

Clinicians must rigorously pursue a preoperative diagnosis, as it may prevent a “surprise” upon laparotomy [17, 18] and result in proper management.

## References

[1] Foo KT, Ng KC, Rauff A (1978). Unusual small intestinal obstruction in adolescent girls: the abdominal cocoon. *British Journal of Surgery*.

[2] Deeb LS, Mourad FH, El-Zein YR, Uthman SM (1998). Abdominal cocoon in a man: preoperative diagnosis and literature review. *Journal of Clinical Gastroenterology*.

[3] Kirshtein B, Mizrahi S, Sinelnikov I, Lantsberg L (2011). Abdominal cocoon as a rare cause of small bowel obstruction in an elderly man: report of a case and review of the literature. *Indian Journal of Surgery*.

[4] Al-Abassi AA, Emad M (2004). Abdominal cocoon. An unusual cause of intestinal obstruction. *Saudi Medical Journal*.

[5] Reynders D, Van der Stighelen Y (2009). The abdominal cocoon. A case report. *Acta Chirurgica Belgica*.

[6] Sieck JO, Cowgill R, Larkworthy W (1983). Peritoneal encapsulation and abdominal cocoon. Case reports and a review of the literature. *Gastroenterology*.

[7] Rao PLNG, Mitra SK, Pathak IC (1979). Abdominal cocoon—a cause of intestinal obstruction in a 4 years old girl. *Indian Pediatrics*.

[8] Rajagopal AS, Rajagopal R (2003). Conundrum of the cocoon: report of a case and review of the literature. *Diseases of the Colon and Rectum*.

[9] Rastogi R (2008). Abdominal cocoon secondary to tuberculosis. *Saudi Journal of Gastroenterology*.

[10] Marinho A, Adelusi B (1980). The abdominal cocoon. Case report. *British Journal of Obstetrics and Gynaecology*.

[11] Seng LK, Mahadaven M, Musa A (1993). Abdominal cocoon: a report of two cases. *British Journal of Surgery*.

[12] Serafimidis C, Katsarolis I, Vernadakis S (2006). Idiopathic sclerosing encapsulating peritonitis (or abdominal cocoon). *BMC Surgery*.

[13] Macklin J, Hall C, Feldman MA (1991). Unusual cause of small bowel obstruction in adolescent girls: the abdominal cocoon. *Journal of the Royal College of Surgeons of Edinburgh*.

[14] Matone J, Herbella F, Del Grande JC (2006). Abdominal cocoon syndrome. *Clinical Gastroenterology and Hepatology*.

[15] Yoon YW, Chung JP, Park HJ (1995). A case of abdominal cocoon. *Journal of Korean Medical Science*.

[16] Kumar M, Deb M, Parshad R (2000). Abdominal cocoon: report of a case. *Surgery Today*.

[17] Chew MH, Sophian Hadi I, Chan G, Ong HS, Wong WK (2006). A problem encapsulated: the rare peritoneal encapsulation syndrome. *Singapore Medical Journal*.

[18] Yip FWK, Lee SH (1992). The abdominal cocoon. *Australian and New Zealand Journal of Surgery*.

